# Serum investigation of antibodies against porcine circovirus 4 Rep and Cap protein in Jiangxi Province, China

**DOI:** 10.3389/fmicb.2022.944679

**Published:** 2022-10-21

**Authors:** Xifeng Hu, Zhen Ding, Yu Li, Zheng Chen, Huansheng Wu

**Affiliations:** ^1^Department of Preventive Veterinary Medicine, College of Animal Science and Technology, Jiangxi Agricultural University, Nanchang, China; ^2^Department of Veterinary Microbiology, Jiangxi Provincial Key Laboratory for Animal Science and Technology, College of Animal Science and Technology, Jiangxi Agricultural University, Nanchang, China

**Keywords:** porcine circovirus 4, ELISA, Cap protein, Rep protein, antibody, serum epidemiology

## Abstract

In 2019, a novel porcine circovirus 4 (PCV4) was first identified in Hunan Province, China. The circular PCV4 DNA was detected in both diseased and healthy pigs. Recently, PCV4 prevalence surveys have been analyzed in many provinces in both China and South Korea with low positive rates. However, no serological data has been conducted to investigate the prevalence of PCV4 in pigs from Jiangxi Province. To address this issue, an indirect anti-PCV4 antibody enzyme-linked immunosorbent assay (ELISA) based on Cap and Rep protein as a coating antigen was established and applied to study the serum epidemiology of PCV4 in Jiangxi Province. Purified PCV4-His-tagged Cap and Rep were used as the coating antigen to develop an ELISA detection kit. There was no cross-reaction of the Cap/Rep-based ELISA with antisera against PCV2, TGEV and PRRSV, indicating a high specificity of this ELISA assay. The intra-assay coefficient variations (CVs) of Cap-based were 1.239%−9.796%, Rep-based 1.288%−5.011%, and inter-assay CVs of 1.167%−4.694% and 1.621%−8.979%, respectively, indicating a good repeatability. Finally, a total number of 507 serum samples were collected from Jiangxi Province to test for antibody prevalence of PCV4, and 17 (3.35%) and 36 (7.10%) of the samples were Cap and Rep antibody positive, respectively. In summary, our established ELISA kit could be used to detect PCV4 antibodies in serum with good repeatability and high specificity. In addition, field samples detection results showed that the antibody of PCV4 was poorly distributed in intensive pig farms in Jiangxi Province, China.

## Introduction

Porcine circovirus (PCV) is a member of genus Circovirus that belongs to the Circoviridae family (Lefkowitz et al., 2018). The virion of PCV is a small, icosahedral and non-enveloped virus containing a single-stranded, closed-circular DNA (ssDNA) (Tischer et al., 1982; Reuter et al., 2014). The length of genome of PCVs is about 1.7–2.0 kb (Tischer et al., 1974). To date, four genotypes of PCVs, PCV1, PCV2, PCV3, and PCV4 have been identified and isolated (Zhang H. H. et al., 2020; Hou et al., 2022). PCV1 was first isolated in cultured porcine kidney (PK-15) cell lines without pathogens (Kim and Chae, 2002; Hirai et al., 2006). Unlike PCV1, PCV2 still poses a threat to the intensive swine industry because PCV2 is the main pathogenic virus causing porcine circovirus-related diseases (PCVAD) (Saporiti et al., 2020; Sibila et al., 2021). PCV3 has been identified in both diseased and healthy pigs (Jiang et al., 2019). In addition, PCV3 has been reported to be associated with porcine dermatitis and nephropathy syndrome (PDNS) (Jiang et al., 2019). From 2019 to 2021, PCV4 was identified as a distinct circovirus species and associated with severe clinical disease with respiratory PDNS via conventional PCR or qPCR in both China and South Korea with low positive rates (Franzo et al., 2020; Ha et al., 2021; Sun R. et al., 2021; Tian et al., 2021; Hou et al., 2022; Kim et al., 2022; Nguyen et al., 2022). Genetically, PCV4 shared the closest relationship and similarity to a mink circovirus with 67% genomic identity, which is higher than PCV1–PCV3 with 52% genomic identity (Zhang H. H. et al., 2020).

Similar to other PCVs, the genome of PCV4 is a 1,770 nt circular single-stranded DNA containing two large open reading frames (ORFs): ORF1 is 891 nt encoding the putative replicase protein (Rep); ORF2 is 687 nt encoding the putative capsid protein (Cap) (Kim et al., 2022). According to a previous report on PCV2, the Cap protein is an important viral affect involved in host cell entry (Cao et al., 2015). The Cap protein mediates the binding of the virus to the cell surface during the invasion process (Ren et al., 2016; Zhang Y. et al., 2020; Sun W. et al., 2021). The literature has shown that the Cap protein plays a crucial biological role in the process of entering host cells via binding to the receptors (Khayat et al., 2011). In addition, the Cap protein is an antigenic epitope that supports the formation of antibodies in the host, thereby stimulating the host to recognize invasive viruses (Kekarainen and Segales, 2015; Park et al., 2021). The Rep protein of PCVs is the major protein that replicates the viral genome and is included in mature virions. The Rep protein is highly expressed during the process of PCV replication, which could promote the production of specific antibodies in the infected host (Hu et al., 2019).

As we all know, a reliable, widely available and highly sensitive and specific serological test for PCV4 specific antibodies could be useful to assess the serological status of pigs, which will provide comprehensive information on the spread and transmission of PCV4. Recently, a variety of methods for the PCV4 Cap protein-based antibody test have been reported and further applied to detect the prevalence of Cap antibodies in Jiangsu Province, China (Lian et al., 2021). Antibody detection of Rep or Cap could help diagnose PCV infection or study the effect of PCV vaccines. Compared to whole virus-based antibody detection methods, purified recombinant protein was used as an envelope antigen to determine the indirect ELISA results to reduce cross-reactivity and improve the accuracy of detection (Lin et al., 2020; Lian et al., 2021). Therefore, both Cap and Rep proteins are one of the most important target proteins for engineered vaccines and detection technologies. In this study, the purified His-Cap or His-Rep proteins were selected as the coating antigens to develop an indirect ELISA to detect PCV4 antibodies. Furthermore, we evaluated the repeatability, sensitivity, and specificity of this indirect ELISA, which was further used to analyze PCV4 antibody levels in intensive pig farms in Jiangxi Province, China. In summary, our study attempted to develop a serological diagnostic method to detect PCV4 infection or to assess vaccine-induced antibody levels in future research.

## Results

### PCV4 Rep and Cap recombinant purification

**Figure 1:**
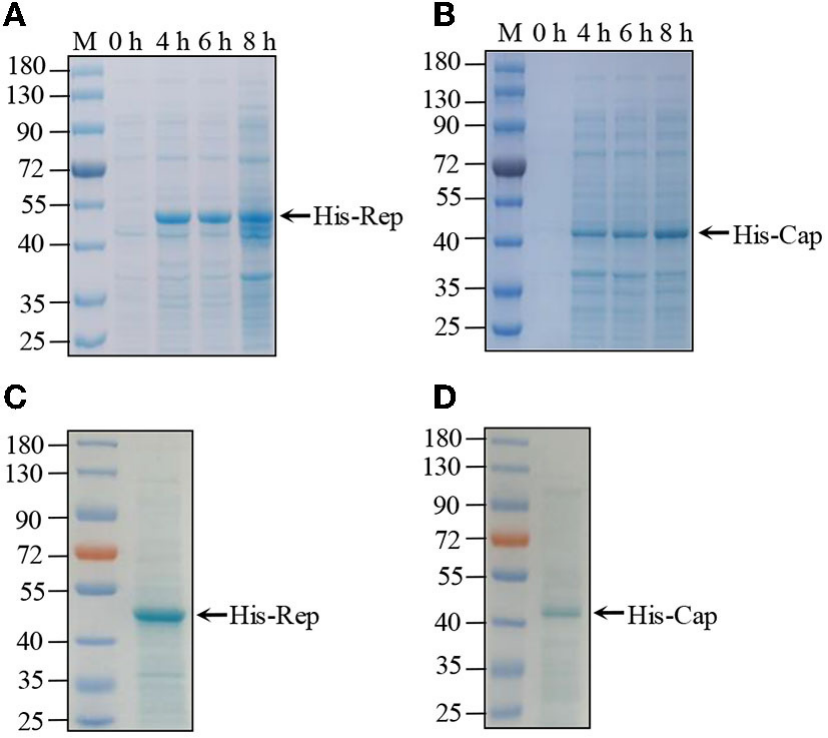
Purification of PCV4 Rep and Cap protein. **(A,B)** His-tagged recombinant protein expression and purification. pET-32a-Rep **(A)** and pET-32a-Cap **(B)** recombinant plasmid individually transferred into BL21pLyss *Escherichia coli* were induced expression by IPTG at different times. Induced protein samples were subjected to SDS–PAGE and Coomassie brilliant blue staining. **(C,D)** Subsequently, expressed His-tagged Rep **(C)** and Cap **(D)** recombinant proteins were purified by Ni-NT assay, which were further analyzed by SDS–PAGE and Coomassie brilliant blue staining.

The complete genome of PCV4 is 1,770 bp that contains two major ORFs, which encodes Rep (891 bp) and Cap (687 bp) proteins (Franzo et al., 2020). Both the Rep and Cap genes were amplified from wild boar samples, which were subsequently cloned into the pET32a vector for induced expression. Recombinant plasmids were transferred into BL21pLyss *Escherichia coli*. The expression of His-Rep and His-Cap proteins was induced expression in the control by IPTG at different times (4, 6, and 12 h). The results shown in Figures 1A,B revealed that both His-Rep and His-Cap were successfully expressed at ~50 and 43 kDa, respectively. Then both His-Rep and His-Cap proteins were further purified by the Ni-NTA method with no obvious non-specific protein determined by Coomassie brilliant blue staining (Figures 1C,D).

### Immunogenicity examination of both Rep and Cap protein

**Figure 2:**
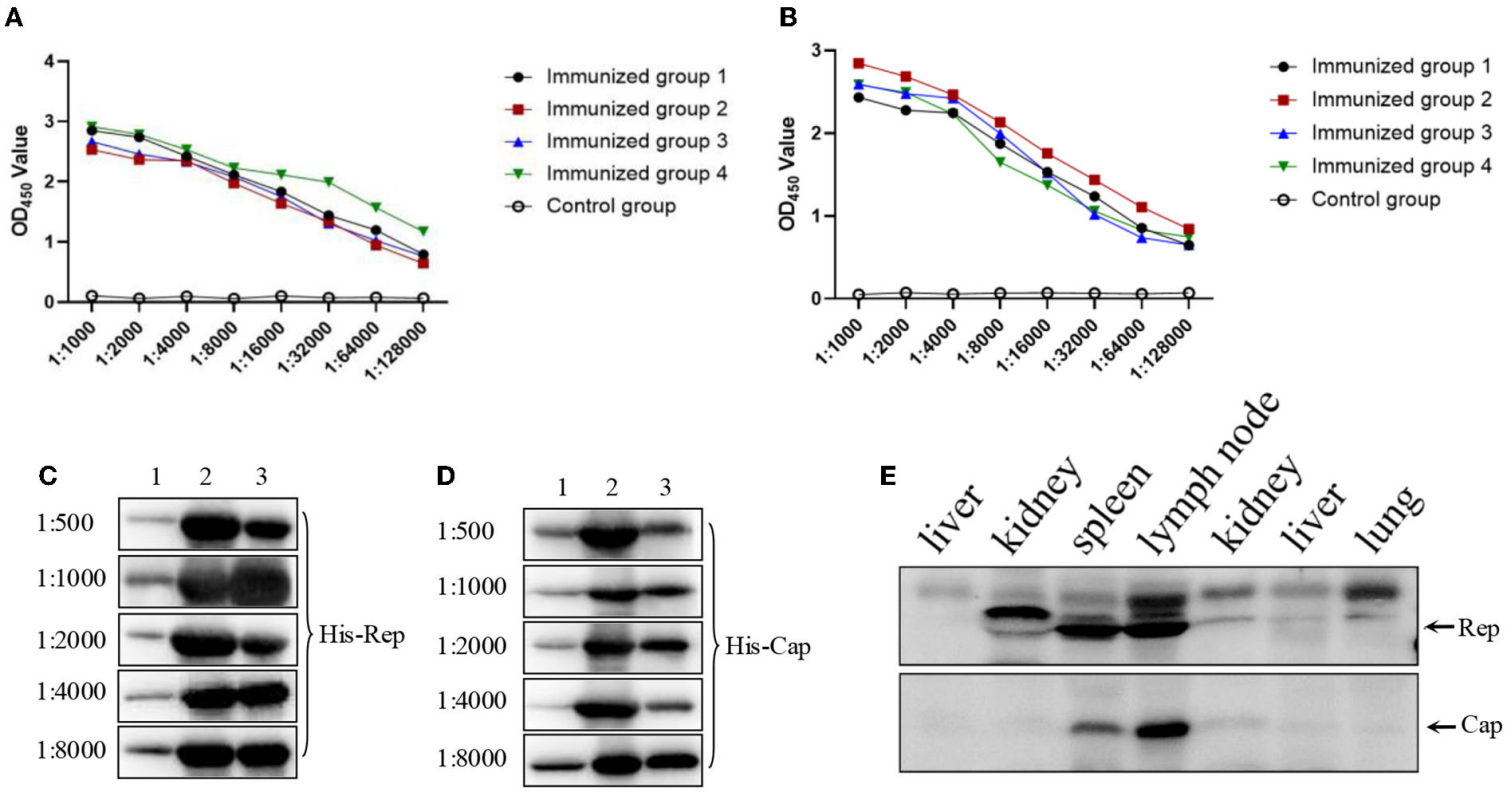
Immunogenicity examination of both Rep and Cap protein. **(A,B)** Four mice antibody titers against Rep **(A)** or Cap **(B)** after third round immunizations. The data were analyzed using Graphpad Prism software 5.0. OD_450_: optical density 450 nm. **(C,D)** Different antibodies against Rep protein **(C)** or Cap protein **(D)** through 1:500 to 1:8,000 were used to react with indicated proteins. The three lanes were as follows: 1, protein expression before induced; 2, protein expression after induced; 3, proteins after purification. **(E)** Both Rep and Cap antibody (1:1,000) were used to detect the endogenous corresponding protein derived from wild boars' tissues including liver, kidney, spleen, lymph node, and lung.

Recombinant His-Cap and His-Rep proteins supplemented with Freund's complete adjuvant were injected individually into four different mice to produce antibodies against Cap and Rep proteins. After the third round of immunization, serums were collected for detection using the established indirect ELISA. Serum antibody titers of Rep and Cap proteins were up to 1:128,000 and 1:64,000, respectively (Figures 2A,B) and were significantly higher than that of control mice. Then the specific antibodies were used to detect the corresponding protein by western blotting with different dilutions. As shown in Figures 2C,D, we found that the 1:8,000 serum dilutions could still react with purified His-Rep and His-Cap proteins. In addition, the positive wild boar samples were additionally detected by Rep and Cap antibodies. We found that both the Rep and Cap antibodies could efficiently react with endogenous viral protein, indicating that the antibody produced could be used for western blotting (Figure 2E), and both Rep and Cap possess good immunogenicity.

### Cut-off value determination of Rep and Cap based ELISA kit

**Figure 3:**
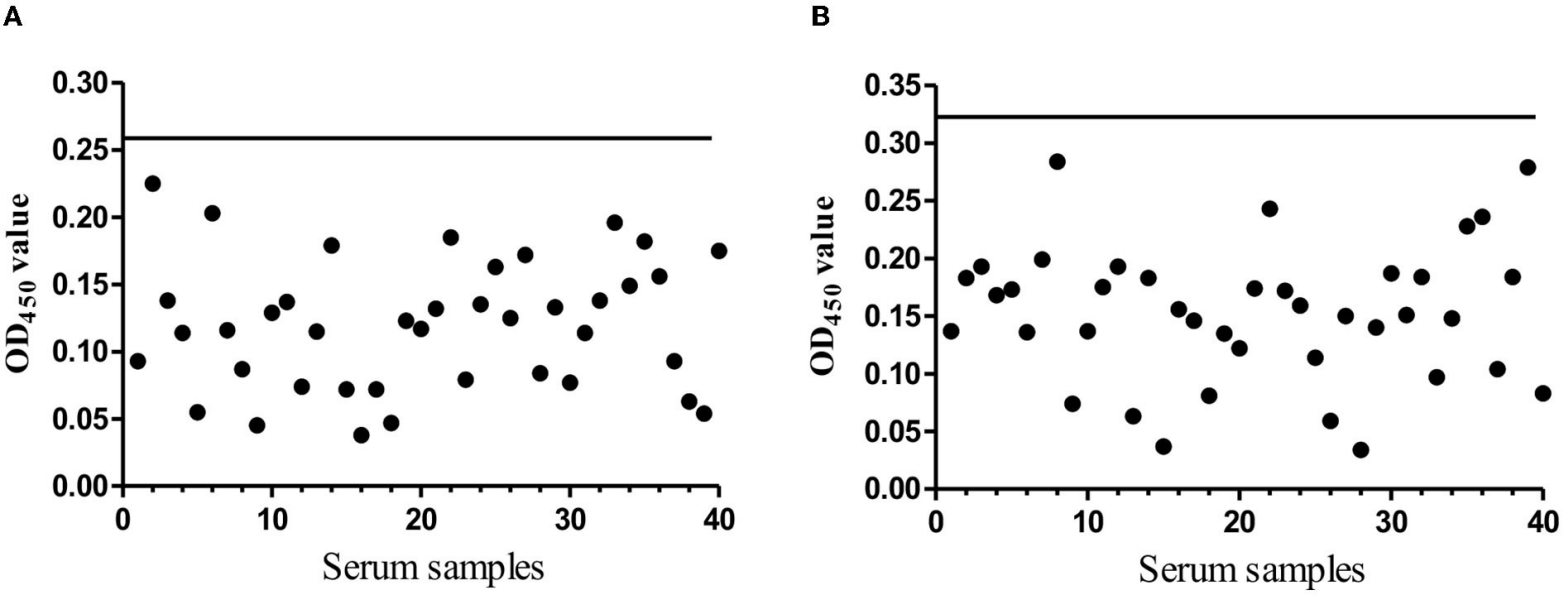
The cut-off value determination. **(A)** The evaluation of the cut-off OD_450_ value of the indirect Rep based ELISA. The black solid line presents the OD_450_ cut-off value (0.262). **(B)** The evaluation of the cut-off OD_450_ value of the indirect Cap based ELISA. The black solid line presents the OD_450_ cut-off value (0.327).

An excellent ELISA kit should have an appropriate cut-off value to characterize the positive or negative sample. To address this issue, the 40 negative serum samples were used to determine the cut-off value. After three independent experiments, both the mean average value (χ) and the standard deviations (SDs) were determined. For Rep-based, ELISA was 0.121 and SD was 0.047. For Cap-based, ELISA was 0.153 and SD was 0.058. The OD_450_ cut-off value was calculated based on the formula OD450 = χ + 3SD, suggesting that the OD_450_ value 0.262 of the samples was recoded as Rep antibody positive; the OD_450_ value 0.327 of the samples as Cap antibody positive was recorded. If not, the serum was considered negative (Figures 3A,B).

### Repeatability of the ELISA kit

In general, an established indirect ELISA kit, such as the commercial ELISA kit, needs a well detectable repeatability. To assess this, we next performed the repeatability determination. Eight negative serum samples were used to examine intra-assay and inter-assay repeatability. As shown in Table 1 (Rep), intra-assay coefficient of variations (CVs) were 1.288%−5.011%, and inter-assay CVs were 1.621%−8.979%. And, as showed in Table 2 (Cap), the intra-assay CVs were 1.239%−9.796%, and the inter-assay CVs were 1.167%−4.694%. Both CVs of the Rep and Cap based ELISA indicate that this indirect ELISA could work very well with excellent repeatability, since CV% values of less than 10% were often recorded as good repeatability.

**Table 1:** Determination of Rep based ELISA coefficient of variation (CV) from eight serums (1–4: positive serum; 5–8: negative serum).

	**Sample**	**χ**	**SD**	**CV%**
Intra-assay	1	1.418	0.018	1.288
	2	0.995	0.014	1.388
	3	1.299	0.019	1.472
	4	1.309	0.026	1.980
	5	0.070	0.003	4.165
	6	0.052	0.003	4.997
	7	0.067	0.003	5.011
	8	0.082	0.002	2.899
Inter-assay	1	1.329	0.023	1.740
	2	1.731	0.028	1.621
	3	1.071	0.029	2.797
	4	1.520	0.028	1.861
	5	0.037	0.003	8.979
	6	0.059	0.005	8.711
	7	0.045	0.003	6.647
	8	0.052	0.003	6.257

**Table 2:** Determination of Cap based ELISA coefficient of variation (CV) from eight negative serums (1–4: positive serum; 5–8: negative serum).

	**Sample**	**χ**	**SD**	**CV%**
Intra-assay	1	1.809	0.022	1.239
	2	1.500	0.068	4.521
	3	1.166	0.029	2.530
	4	1.007	0.027	2.679
	5	0.069	0.007	9.796
	6	0.058	0.002	4.129
	7	0.059	0.004	6.357
	8	0.063	0.003	5.209
Inter-assay	1	1.145	0.054	4.694
	2	1.208	0.052	4.321
	3	1.448	0.024	1.647
	4	1.720	0.020	1.167
	5	0.199	0.004	1.859
	6	0.059	0.002	3.556
	7	0.124	0.005	3.863
	8	0.124	0.002	2.250

### Sensitivity and specificity analyzing of the indirect ELISA

**Figure 4:**
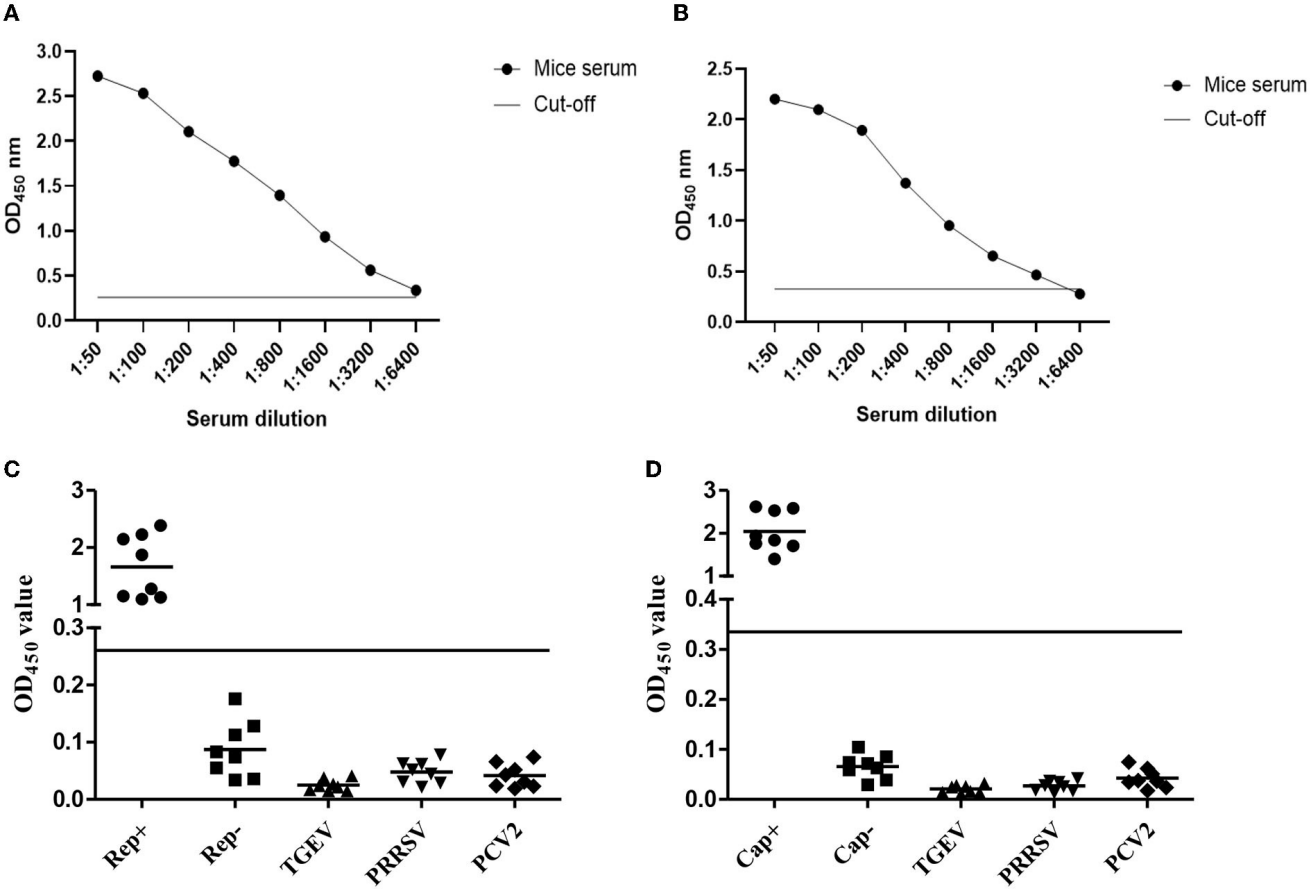
Sensitivity and specificity analyzing of the indirect ELISA. **(A,B)** Antibody against Rep protein **(A)** or against Cap protein **(B)** diluted from 1:50 to 1:6,400 was individually used to test the sensitivity of ELISA. **(C,D)** Eight indicated serum samples were used to analyze the specificity of Rep-based **(C)** and Cap-based **(D)** ELISA. The OD_450_ value higher than the value of black line was recorded as positive, if not, negative.

As we all know, a well-functioning, well-established indirect ELISA kit must be free of non-specific reactions as well as being highly sensitive. To determine the sensitivity of the Rep- or Cap-based ELISA method, the dilutions of Rep antibody from 1:50 to 1:6,400, of Cap antibody from 1:50 to 1:32,00 were used to determine the Rep- Detect protein or the Cap protein. The Rep OD_450_ value of 1:6,400 is 0.285 (Figure 4A) and the Cap OD_450_ value of 1:3,200 is 0.298 (Figure 4B). 1:3,200 serum dilutions are sensitive to Rep- or Cap-based ELISA detection. To address this problem of non-specific reactions, eight serum samples each positive for Rep or Cap, negative for Rep or Cap, TGEV, PRRSV, and PCV2 were used to analyze the specificity of this indirect ELISA. The results showed in Figures 4C,D suggest that indirect ELISA cannot cross-react with antisera to other pathogens or PCV4-negative serum samples.

### Application of ELISA in testing antibodies in field samples

Finally, the ELISA kit should be used to detect the antibodies against PCV4 Rep and Cap protein in field samples. A total number of 507 field serum samples collected from intensive pig farms in Jiangxi Province were used to detect the antibodies. The results shown in Tables 3, 4 showed that 36 (7.10%) of the samples were positive for Rep antibody and 17 (3.35%) of the samples were positive for Cap antibody. Among the serum samples, 12 (11.76%) of the samples were positive for Rep antibody from JiAn, which was higher than those from other pig farms including Nanchang (7.92%), Shangrao (2.97%), Yichun (7.92%) and Ganzhou (4.90%), indicating that the rate of Rep antibodies was low in positive mice. The low Rep antibody positive rate was remarkably lower than the 43.97% positive rate reported in previous data. However, the low Cap antibody positive rate in this study was almost equal to that in Jiangsu Province (3.44%). Thus, field serum samples detected by established Rep-based and Cap-based ELISA showed that both Rep and Cap antibodies were distributed at low rates in Jiangxi province. Nevertheless, our limited serum samples may not reflect the average antibody distribution in our country, necessitating extensive studies of PCV4 serum epidemiology.

**Table 3:** The Rep antibody detection results by the ELISA from 507 serum samples.

	**Samples** ** (*n*)**	**Positive** ** (*n*)**	**Negative** ** (*n*)**	**Positive** ** rate (%)**
NanChang	101	8	96	7.92
ShangRao	101	3	98	2.97
JiAn	102	12	90	11.76
YiChun	101	8	93	7.92
GanZhou	102	5	97	4.90
Total	507	36	471	7.10

**Table 4:** The Cap antibody detection results by the ELISA from 507 serum samples.

	**Samples** ** (*n*)**	**Positive** ** (*n*)**	**Negative** ** (*n*)**	**Positive** ** rate (%)**
NanChang	101	5	96	4.95
ShangRao	101	3	98	2.97
JiAn	102	1	101	0.98
YiChun	101	6	95	5.94
GanZhou	102	2	100	1.96
Total	507	17	490	3.35

## Discussion

Recently, porcine circovirus 4 (PCV4) has been identified in several provinces in China, including Jiangsu, Henan, Hunan, Guangxi, Fujian, Anhui and Shanxi, and in South Korea (Lian et al., 2021). The total length of the PCV4 genome is 1,770 bp. Although a Taq-Man based qPCR assay could be used to detect PCV4 infection, there is no suitable method to detect PCV4 antibodies. In addition, there is no serological epidemiology of PCV4 in Jiangxi Province. Therefore, in this study, we developed an indirect ELISA method to analyze antibodies against PCV4 Rep and Cap protein because the ELISA assay is inexpensive, highly specific, and suitable for large-scale detection of serum samples. In addition, it was reported that the sensitivity of Rep-based ELISA was lower than that of Cap-based ELISA for PCV2 detection. And there is little identity of both Rep and Cap proteins between PCV4 and other PCVs, indicating that there may not be any cross-reactivity with other PCVs, consistent with our data in this study.

In this study, both the PCV4 Rep and Cap proteins were delivered to the prokaryotic expression system in *E. coli*. First, we obtained the full length Rep and Cap genes inserted into the expression vector. After induced expression by IPTG purified by Ni-NTA, SDS–PAGE results showed that the expression and purification of His-Rep and His-Cap proteins were as effective as expected. Furthermore, the enhanced immunogenicity after the third round showed that the specific antibodies produced by injected mice could efficiently react with His-Rep and His-Cap proteins but not with TGEV, PRRSV, and PCV2. An excellent ELISA kit must require well-functioning repeatability, high sensitivity, and high specificity. To clarify the characteristics of this ELISA assay, the repeatability evaluation showed that the value of both intra-assay and inter-assay was < 10%, suggesting that this Rep-based and Cap-based ELISA kit base with good repeatability worked (Mu et al., 2021).

Usually, the diagnosis of a viral infectious disease requires the detection of antigens (or viral nucleotides) or antibodies, both of which can help characterize the prevalence of pathogens (Han et al., 2021).

At present, there is no PCV4 vaccine application in China, leading to the urgent need to introduce ELISA to detect the antibody level of PCV4. In this study, a total number of 507 serum samples from intensive pig farms in Jiangxi Province were collected for field sample detection. The data showed that 7.10% (36/507) of the samples were PCV4 Rep positive and 3.35% (17/507) of the samples were PCV4 Cap positive, indicating a low prevalence of PCV4 in pigs. This low positive rate of both Rep and Cap antibodies of PCV4 was also found in Jiangsu Province. Still, according to a previous report, there was a high positive rate of 43.97% in 17 provinces in China. The high difference in the positive rate could be due to the different detection method used by different study farms, which needs further investigation. However, there were several limitations in this study. (1) Since the pathogenicity of PCV4 has not been clarified to date, the serum samples taken were not focalized, resulting in the low positive rate of PCV4 antibodies cannot reflect a threat of PCV4 infection; (2) In general, a new indirect ELISA-based detection kit needs to be validated by comparison with the same or a similar commercial ELISA kit. Although the primary application of this novel ELISA kit can potentially be used as a diagnostic test, further comparative analyses of this indirect ELISA still need to be performed. (3) A small number of serum samples from a limited area restricted the application of this novel ELISA kit, the low PCV4 positive rate in Jiangxi province did not reflect the positive rate in our country. Therefore, large-scale serum antibody studies must continue to be performed.

## Materials and methods

### Serum samples collection

A total number of 507 pig serum samples were collected from different intensive pig farms located in Nanchang, Shangrao, JiAn, Yichun and Ganzhou in Jiangxi Province, China, between January 2021 and December 2021. The detailed information of serum samples showed in Tables 3, 4. The Committee on the Ethics of Animal Experiments of Jiangxi Agricultural University supervised the serum samples collection.

### PCV4 Cap and Rep protein expression

In order to obtain the full-length Cap and Rep gene of PCV4, the complete genome of strain JXWY-2020 (MW988108) was first obtained. The Cap and Rep genes were then inserted into pET-32a-vector after digestion with XhoI and EcoRI and T4 ligation. Finally, the recombinant plasmid was transferred into competent *E. coli* BL21 pLyss cells. Subsequently, both His-Cap and His-Rep proteins were expressed under the control of 2.0 mM IPTG at 37 for 8 h. Expressed recombinant protein was further analyzed by SDS–PAGE and Coomassie brilliant blue staining. Finally, both recombinant proteins were purified through a Ni-NTA affinity chromatography column in non-denaturing state based on the Ni-NT purification kit instructions.

### Immunogenicity determination

About 20–25 g, six-week-old BALB/c mice purchased from the Center for Experimental Animals of Jiangxi Traditional Medical University were kept under normal conditions in the animal care facility of Jiangxi Agricultural University. Both purified recombinant His-Cap and His-Rep protein (30 g/mouse) emulsified with Freund's complete adjuvant were injected individually into four different mice (one mouse was used as a negative control). After 1 week, and another 2 weeks later, these mice were immunized a second and third time individually with 30 and 200 g/mouse. After another week, we carried out the fourth immunization with injection of 100 g/mice accompanied by complete Freund's adjuvant. The immunized serum was collected from the caudate artery. Finally, the immunized mice were euthanized in a CO_2_ chamber. The antibody of Cap and Rep was determined using the established ELISA in this study to assess antibody titer.

### Indirect ELISA for analyzing both anti-Cap and anti-Rep antibodies

According to the guideline of the P/N value, the perfectly suitable conditions of the indirect ELISA have been optimized, such as blocking solution, sera, dose of coated antigen protein, concentrations of HRP-conjugated goat anti-pig IgG and its reaction time. After process optimization, we performed the following best reaction conditions: 1.25 mg/ml His-Rep and 2.5 mg/ml His-Cap were suitable for loading onto the plate. The coated plates were washed three times with PBST, then 100 μl of 1% bovine serum albumin (BSA) was added to each well and incubated at 37°C to block for 1.5 h for Cap protein; 100 μl of 5% milk was added to each well and incubated at 37°C for blocking for 2 h for Rep protein. Fifty liters of serum diluted 1:800 was added to react at 37°C for an additional hour. Finally 100 μl TMB substrate solution and 10 min incubation at room temperature stopped by loading 100 μl ELISA final solution buffer.

### The cut-off value determination

To confirm the OD_450_ value, 40 negative serum was used to detect three times. For Rep protein: The standard deviation (SD) was 0.047 and the mean PR value (χ) was 0.121. The cut-off value of OD_450_ is in the conversion based on the following formula: OD_450_ value = χ + 3 SDs = 0.262. Finally, the OD_450_ ≥0.262 was recorded as a positive sample, if not, it was negative. For Cap protein: SDs was 0.058, χ was 0.153, and OD_450_ ≥0.327 was recorded as a positive sample, if not, it was negative.

### The examination of coefficient of variations

To further assess CVs, four positive and negative serum samples were used to assess both intra-assay and inter-assay variations of Rep and Cap proteins. The value of each sample was displayed as mean, standard deviation, and CVs. Intra-assay CVs were determined from the results of the quadruple detection using the same procedure. In addition, inter-assay CVs were calculated based on the result of four-time analysis on different days within the same assay.

### Sensitivity and specificity analyzing of the indirect ELISA

To determine the sensitivity of the Rep- or Cap-based ELISA method, the dilutions of Rep antibody from 1:50 to 1:6,400, and of Cap antibody from 1:50 to 1:3,200, were used to determine the Rep-Detect protein or the Cap protein. The Rep OD_450_ of 1:6,400 is 0.285 and the Cap OD_450_ of 1:3,200 is 0.298, which is closest to the cut-off OD_450_, indicating that the 1:6,400 or 1:3,200 dilutions of serum most sensitive are Rep or Cap based ELISA detection. In addition, to assess the specificity of the indirect ELISA, eight serum samples each against PCV2, PPRSV, TGEV, and negative serum against PCV4 Rep or Cap were used to assess its specificity within the same ELISA procedure with triplicate experiments and the OD_450_ value was used to characterize whether the sample was positive or negative.

### Antibodies of field samples detection

A total number of 507 serum samples were collected from intensive pig farms in Nanchang, Shangrao, JiAn, Yichun and Ganzhou in Jiangxi Province, China. All the serum samples were detected according to the protocols in this indirect ELISA kit. The OD450 value ≥0.262 of serum samples was recorded as a positive sample of Rep antibody, if not, it was negative sample. The OD450 value ≥0.327 of serum samples was recorded as a positive sample of Cap antibody, if not, it was recorded as negative.

## Data availability statement

The original contributions presented in the study are included in the article/supplementary material, further inquiries can be directed to the corresponding author.

## Ethics statement

The animal study was reviewed and approved by the Committee on the Ethics of Animal Experiments of Jiangxi Agricultural University.

## Author contributions

HW conceived and designed the experiments. XH, ZD, and YL performed the experiments and analyzed the data. HW, ZD, and ZC wrote, reviewed, revised, and edited the manuscript. All authors have read and agreed to the published version of the manuscript.

## Funding

This work was supported by grants from the Natural Science Foundation of Jiangxi Province (20202BABL215023) and the Jiangxi Provincial Department of Education (GJJ190213).

## Conflict of interest

The authors declare that the research was conducted in the absence of any commercial or financial relationships that could be construed as a potential conflict of interest.

## Publisher's note

All claims expressed in this article are solely those of the authors and do not necessarily represent those of their affiliated organizations, or those of the publisher, the editors and the reviewers. Any product that may be evaluated in this article, or claim that may be made by its manufacturer, is not guaranteed or endorsed by the publisher.
